# On the Use of the Fixed Alkali in the Hooping Cough

**Published:** 1803-02-01

**Authors:** 


### *On the Use of the fixed Alkali in the Hooping Cough; communicated by Dr.* NOEHDEN.

THE fixed vegetable alkali was first recommended in this disease by Dr. Stutz, whose observations on the use of that remedy, in several nervous disorders, were some time since communicated to the public through the medium of Hufeland's Journal. Dr. Memminger, of Reutlingen, is the first who successfully tried the fixed alkali in an epidemic hooping cough, which had frequently frustrated the known methods of cure. The following is the mode of treatment which he at last adopted. In the beginning he gave every two or three days, one after another, *Syrup Ipecacuanha of de Lassone* with *Vin. Antimon. Huxham,* to be taken by tea spoons, till it had produced vomiting three or four times. He then prescribed, according to the different ages of the children, from four to twelve grains of *Alkali fix.* in *Aq. Cinam. S. V.* with *Syrup. Cortic. Aurantior*; besides which, he ordered *Laud. Liq. Sydenh* to be taken three times a day, in a dose adapted to the different ages of the children. Warm baths, clysters, and the rubbing in of *Linn. Volat.* with *Tinct. Cantharid.* were used besides, as auxiliary remedies; and in the third stage of the disease the extract chinæ was given. In order to ascertain what was the effect of the alkali, when in combination with laudanum, and when given by itself, he used it without employing any other remedy, except a previous emetic, and he had the satisfaction to find, that it proved very efficacious; the most obstinate cough, which frequently lasts for three or four months, being moved by it within five or six weeks, without any ill consequences.

CRITICAL

